# Residence time, native range size, and genome size predict naturalization among angiosperms introduced to Australia

**DOI:** 10.1002/ece3.3505

**Published:** 2017-10-27

**Authors:** John P. Schmidt, John M. Drake, Patrick Stephens

**Affiliations:** ^1^ Odum School of Ecology University of Georgia Athens GA USA

**Keywords:** Bayesian network, comparative study, genome size, invasive plants, phylogenetic logistic regression, polyploidy, residence time

## Abstract

Although critical to progress in understanding (i) if, and (ii) at what rate, introduced plants will naturalize and potentially become invasive, establishing causal links between traits and invasion success is complicated by data gaps, phylogenetic nonindependence of species, the inability to control for differences between species in residence time and propagule pressure, and covariance among traits. Here, we focus on statistical relationships between genomic factors, life history traits, native range size, and naturalization status of angiosperms introduced to Australia. In a series of analyses, we alternately investigate the role of phylogeny, incorporate introduction history, and use graphical models to explore the network of conditional probabilities linking traits and introduction history to naturalization status. Applying this ensemble of methods to the largest publicly available data set on plant introductions and their fates, we found that, overall, residence time and native range size best predicted probability of naturalization. Yet, importantly, probability of naturalization consistently increased as genome size decreased, even when the effects of shared ancestry and residence time in Australia were accounted for, and that this pattern was stronger in species without a history of cultivation, but present across annual–biennials, and herbaceous and woody perennials. Thus, despite introduction biases and indirect effects of traits via introduction history, across analyses, reduced genome size was nevertheless consistently associated with a tendency to naturalize.

## 1 INTRODUCTION

Within the field of invasion biology, predicting which introduced species will successfully naturalize, and at what rate, motivates a large body of research. A common set of traits that are often associated with invasion success, measurable prior to species introductions, appears to be identifiable (e.g., Pyšek et al., 2009). Candidate traits enable effective screening (Schmidt, Springborn, & Drake, 2012; Schmidt, Stephens, & Drake, 2012) and may form the foundation for a detailed mechanistic theory of the eco‐evolutionary drivers and controls on the spread of species into new communities, ecosystems, and regions. In practice, however, the biology of species can be difficult to disentangle from variables that Peoples and Goforth (2017) call “event‐level” factors: propagule pressure (i.e., the number of individuals released, the degree of effort toward propagation by humans, Lockwood, Cassey, & Blackburn, 2005) and time since introduction. Features of the introduced range, such as biotic resistance and climate matching, are also important determinants of successful invasions. Predictive models of invasion success have most often considered traits without being able to control for event‐level factors (Keller, Kocev, & Džeroski, 2011). When incorporated into models, the magnitude of the effect of event‐level factors has typically been much greater than the effect of traits (Peoples & Goforth, 2017). Furthermore, species‐level and event‐level factors are unlikely to be independent so that traits may influence naturalization indirectly via biases in introductions (Maurel, Hanspach, Kühn, Pyšek, & van Kleunen, 2016), use, and intensity or geographic scale of cultivation.

In the case of flowering plants, polyploidy and genome size (see reviews by te Beest et al., 2012; Suda, Meyerson, Leitch, & Pyšek, 2014) have been proposed as key features influencing the successful colonization and spread of alien plants (Pandit, White, & Pocock, 2014). Polyploidization (whole genome duplication) can promote rapid evolution in angiosperms (Leitch & Bennett, 1997; Soltis & Soltis, 2000). Genome size, which is positively correlated with generation time and seed mass, but negatively with relative growth rate, may serve as a master trait that affects several components of fitness (Suda et al., 2014). However, the advantages of the polyploidization and reduced genome sizes are likely to be strongly mediated by performance‐related functional traits (e.g., seed mass, plant height, woodiness and wood density, and leaf longevity) and associated life history patterns. For example, genome size and rates of polyploidization are known to differ by growth form (Knight, Molinari, & Petrov, 2005; Leitch & Leitch, 2012; Stebbins, 1938) such that both annual plants and woody species tend toward smaller genome sizes than herbaceous perennials, and polyploid cytotypes are more rare in woody than herbaceous plants. As species with rapid generation times (annuals and biennials, herbaceous species) have been shown to make up a higher proportion of naturalized species than slower‐maturing perennial and woody species (Caley, Groves, & Barker, 2008; Kloot, 1986), controlling for residence time is critical for detecting whether and how the effect of genomic factors influences naturalization across growth forms.

Questions related to the eco‐evolutionary processes underlying plant invasions are frequently investigated by means of comparative studies. Comparative approaches allow for broad generalizations that controlled field experiments on a few taxa cannot. However, the robustness of findings from comparative analyses is often compromised by significant data gaps. Lists of species that have been introduced but have not naturalized or become invasive are typically incomplete (Bucharova & van Kleunen, 2009; Reichard & Hamilton, 1997; Schmidt & Drake, 2011) so that introduction failures are poorly documented relative to successes. Owing to the lack of data, differences in residence time and propagule pressure in exotic ranges often cannot be controlled for (Pyšek & Jarošík, 2005; Schmidt & Drake, 2011). Moreover, while capturing phylogenetic dependencies among taxa may sometimes be critical to reliably interpreting the statistical effects of traits on outcomes, both developing phylogenetic trees for estimating relatedness across taxa in large data sets (Felsenstein, 1985) and incorporating the associated complex error structures into statistical models (e.g., Ives & Garland, 2010; Revell, 2010) remain methodological and computational challenges. For all of these reasons, phylogenetically controlled comparative studies that include the introduction histories of species are scarce in the literature on biological invasions (Pyšek et al., 2009, 2015).

In the present study, we combine information on earliest collection dates and cultivation status with data on genomic and functional traits and native range size to statistically model the naturalization status of species introduced to Australia. Our goal is to understand how genomic and morphological features of angiosperms contribute to successful invasions. Interpreting statistical results as information about which factors may be key drivers of invasion success is complicated when candidate predictors are correlated, and when the availability of trait data is biased (here, toward cultivated species and economically damaging invasive species). Therefore, using the largest publicly available data set on plant introductions (Randall, 2007), we adopt a broad inferential framework that relies on a series of statistical approaches (graphical models, phylogenetic generalized linear models, survival analysis, and generalized additive models) to identify the direct and indirect causal linkages between the biology of individual species and successful naturalization.

## 2 METHODS

### 2.1 Naturalization status

Randall (2007) compiled a list of 26,242 plants introduced to Australia and the current status of each one as naturalized or invasive. We then harmonized Randall's taxonomy with that of The Plant List (http://www.theplantlist.org/) to create one of the largest databases of its kind. The Randall database labels each introduced species as an “escape from cultivation”, “naturalized”, an “agricultural weed” (AW), “natural area invader” (NAI), “noxious” (a species with some legal restriction), “invasive” (a species causing significant environmental and/or economic damage), or not naturalized (to date). While obviously coarse compared to direct quantitative measures of invasion success such as invasive range size or dominance of native plant communities, such data would have been available only for a small fraction of the species we included sharply limiting the potential geographic scope of our analyses. In Randall's database, species frequently have multiple labels corresponding to different regions and political units within Australia. For our analyses, we ignored the noxious designation (because a legal rather than ecological category) and chose the most extreme label assigned to each species based on the ordering introduced<escape<naturalized<AW<NAI<invasive. This progression roughly corresponds to average time since earliest Australian herbarium record (hereafter “residence time”) available from Australia's Virtual Herbarium (AVH 2015), with mean residence times of 49.2, 46.4, 78.3, 80.3, 92.5, and 69.0 years, respectively (Table 1). Using this scheme, we classified all species labeled as naturalized, AW, NAI, or invasive somewhere in Australia as naturalized, and the remainder of species as not naturalized.

**Table 1:** Summary statistics for age of earliest Australian herbarium record by naturalization status of angiosperms introduced to Australia (Randall, 2007) for which herbarium records were available from Australia's Virtual Herbarium (AVH 2015)

Status	*n*	Median residence time	Mean residence time	SD	Max	Min
Introduced only	2,314	36	49.2	40.4	245	1
Escape from cultivation	17	46	46.4	29.9	112	12
Naturalized	865	76	78.3	45.1	245	2
Agricultural weed	143	83	80.3	44.6	245	2
Natural area invader	1,648	99	92.5	43.2	245	2
Noxious	118	86	80.8	40.0	213	2
Invasive	19	65	69.0	40.4	154	6
Total	5,125					

### 2.2 Covariate traits

For species in Randall's list, we compiled data on genomic and functional traits as well as native range size and introduction history. Table 2 provides a summary of covariates and sample sizes. We obtained genome size data from the Royal Botanic Garden Kew Database (Bennett & Leitch, 2012; http://data.kew.org/cvalues/). In the Kew database, the majority of C‐values (genome size quantified as picograms of DNA present in the haploid nucleus of a eukaryotic organism) were accompanied by ploidy number to indicate whether the cytotype was, for example, diploid (2n) or tetraploid (4n). Because many species have multiple cytotypes and thus multiple entries in the Kew database, we divided *C*‐value by the ploidy number given in the database to calculate monoploid (Greilhuber, DoleŽel, Lysak, & Bennett, 2005) genome size. The resulting values were very similar for genome size across cytotypes within the same species. We also included largest holoploid genome size—the DNA content of the entire chromosome complement of the individual organism—for the highest ploidy level for each species in the Kew database. We restricted *C*‐value data to prime entries that also included ploidy number or chromosome counts. Monoploid values ranged from 0.08 to 76 picograms, and holoploid values from 0.18 to 254.8 picograms. We used data from the Index to Plant Chromosome Numbers (IPCN; http://www.tropicos.org/Project/IPCN) to identify species with multiple cytotypes, indicating recent polyploid or aneuploid events, and used this determination to create a binary variable reflecting whether multiple cytotypes were known for each species.

**Table 2:** Sample sizes of all covariates, and units, ranges, means, and standard deviations of continuous covariates

Trait	*n*	Max	Min	Mean	SD
Ploidal variation	9,341				
Multiple cytotypes	2,371				
Single cytotype	6,970				
Largest holoploid genome size (picograms)	3,008	254.8	0.18	4.9	3.8
Monoploid genome size (picograms)	2,210	76.0	0.08	1.9	3.9
Life history and growth form	13,544				
Woody	7,607				
Herbaceous perennial	4,813				
Annual/biennial	1,124				
Maximum height (m)	1,813	70	0.03	1.6	4.4
Seed mass (g)	3,038	631	3 × 10^−6^	0.3	1.5
Native range size (number of ecoregions)	26,619	1	33	1.1	1.4
Propagule pressure	26,619				
Cultivated	3,624				
Not cultivated	22,995				
Earliest herbarium record (years)	5,125	245	1	51	2

To characterize functional trait differences among species, we selected a group of traits—native range size, seed mass, maximum height, and woody versus herbaceous—which have repeatedly been identified as significant predictors of naturalization in previous studies, and for which data are readily available for a large pool of species (Pyšek & Richardson, 2008; Pyšek et al., 2009; Schmidt, Springborn et al., 2012; Schmidt, Stephens et al., 2012). We obtained values for average seed mass, which ranged from 0.3 μg to 631 g (mean = 0.3 mg, SD = 1.5 mg) from the Royal Botanic Garden Kew Seed Information Database (http://www.kew.org/data/sid), and for mature height, which ranged from 0.03 to 52.5 m (mean = 1.6 m, SD = 4.4 m), from the Plants National Database (USDA NRCS, http://plants.usda.gov/), the TRY Plant Trait Database (http://www.try-db.org/TryWeb/), and horticultural sources such as PlantFiles (http://davesgarden.com/guides/pf/). From the sources above, we also compiled data on whether species can occur as annuals, biennials, and as woody or herbaceous perennials. The Germplasm Resources Information Network (GRIN 2015) divides the globe into 52 floristic regions (Brummitt, Pando, Hollis, & Brummitt, 2001). Each species covered by the GRIN database is recorded as present in a subset of these regions. For each species in our data set, we summed the number of floristic regions in which the species is known to be present as a native. This offers a rough measure of native range size, but also environmental tolerance, and was an important predictor in a previous comparative study (Schmidt, Springborn et al., 2012; Schmidt, Stephens et al., 2012). Native range size values ranged from 1 to 33, although 3,948 of 5,125 species, or 77%, were known from a single ecoregion only.

For a subset of species, we estimated residence time (conservatively) in Australia by subtracting the date of the earliest dated herbarium specimens provided by Australia's virtual herbarium (AVH 2015) from the year Randall's database was published (2007). Whereas any given species could potentially have been introduced in Australia well prior to initial collection, these records do provide a useful measure of the minimum amount of time that species may have been present. In addition, from data provided by AVH documenting species known to have been cultivated historically, we labeled species as cultivated or not. To improve the fit of models in the analysis of the data compiled and to facilitate visualization of data and patterns, all covariates were normalized by log_10_‐transformation.

### 2.3 Bayesian network

Ecological and life history variables are not causally independent but exist in a complex web of interconnections. This complicates the interpretation of regression analyses in large comparative studies, particularly if the goal is to generate or test hypothetical causal mechanisms. Bayesian networks offer a statistical approach for handling and visualizing probabilistic dependencies between variables. In a Bayesian network, variables are presented graphically as nodes that can serve as “parents” via conditional probabilities to other “child” nodes (individual nodes can occupy both positions). Arrows (edges) indicate these conditional probabilities or dependencies between parent and child nodes (Korb & Nicholson, 2010). Because the network is learned directly from the data with minimal prior assumption, Bayesian networks are useful for inferring causal relationships from variables that are conjectured to covary.

To learn the network structure, we used log‐transformed covariates to normalize their distributions and performed Tabu Search (Glover, 1986), a modified hill‐climbing algorithm, using the bnlearn package (Scutari, 2010) in R (R Core Team, 2016) on a complete case subset of the data (*n *=* *688, NAI* *= 413, AW = 432) for the following covariates intended to parsimoniously capture key features of life history and the introduction histories of species: time from earliest Australian herbarium record, whether known to have been cultivated, native range size, seed mass, mature height, whether woody, whether annual, whether multiple cytotypes have been reported, monoploid and largest holoploid genome sizes. We then fit parameters to the network conditional on its structure. A strength of bnlearn is that networks can be learned via any of a suite of algorithms. Although results were qualitatively similar across many algorithms, we chose Tabu Search—rather than constraint‐based or local discovery algorithm—because it offers a good trade‐off between computational demands and the accuracy of the models learned (Gamez, Mateo, & Puerta, 2011). Applying these methods, we developed a Bayesian network in which AW, NAI, and “naturalized” (naturalized without having been labeled as either AW or NAI by Randall) were the classes to be predicted. Aside from disallowing these categories as parent nodes or predictors of one another, we made no prior restrictions on connectivity or direction of edges. Initially, three classes (naturalized, AW, and NAI) were used. Because connecting edges and their strengths were nearly identical in the case of NAI and naturalized classes, we then combined the two classes to simplify the network and its interpretation. We also tested for the potentially confounding effect of shared ancestry among species on the structure of the network model and found phylogenetic effects not to be important (Appendix I). Resulting models thus visualize which factors or traits directly linked by conditional probabilities to either naturalized class and the magnitude and sign of each link, but also reveal causal relationships that are joint or indirect or diffuse by similarly diagramming relationships among all the covariates in the model.

### 2.4 Univariate regression analysis

To complement Bayesian network analysis and to make the best inferential use of the data set we compiled, we also investigated univariate relationships between traits and whether naturalized using phylogenetic generalized least‐squares analyses. We report methods and results of these analyses in the Appendix S1.

To estimate variance explained by each trait in univariate regressions, we performed logistic regression without controlling for phylogeny using *glm* in R. We then used Nagelkerke's pseudo‐*r* ^2^ (Nagelkerke, 1991) to assess model fit.

### 2.5 Survival analysis

We performed univariate survival analysis to study the effect of residence time on naturalization status while controlling for traits and historical factors. Survival analysis is a statistical method for analyzing the expected duration of time until an event, in the present case successful naturalization of an introduced species, occurs. Importantly, survival analysis readily accommodates censoring, a form of missing data in which exact dates are unknown but a set of temporal bounds exist. In the case of invasive status, we derived a rough estimate for year of introduction from the year of the first herbarium record, but the date when a species was considered naturalized was not available. Therefore, naturalization status is interval‐censored—the interval being one extending from the date of introduction to 2007. For species not classified as naturalized, the data are right‐censored to the extent that many species that have not naturalized so far may eventually do so (Caley et al., 2008).

In proportional hazards models, one particularly general class of survival models, hazard is defined as the rate that events occur at time *t* given survival until time *t*, and the covariate is multiplicatively related to hazard. For continuous covariates, the hazard responds logarithmically so that each unit increase in the covariate results in proportional scaling of the hazard. We estimated the Cox Proportional Hazards model (David 1972) with the survival package (Therneau, 2015) in R using the *pspline* command to incorporate potentially nonlinear responses into survival models. We generated predictions for continuous variables as hazard rates (i.e., rate of naturalization) and compared the rates of cultivated to uncultivated species. For categorical predictors, we visualized differences between classes (annuals/biennials, herbaceous perennials, woody, and multiple versus single cytotype) using Kaplan–Meier plots of the probability naturalized as a function of time.

### 2.6 Genomic factors and growth form

To test whether the distribution of genome size differs by naturalization status and growth form, we compared the frequency of species with multiple cytotypes using contingency analysis with a conservative, Bonferroni, correction (Hochberg, 1988) for multiple comparisons, and the means and medians of largest holoploid and monoploid genome size by growth form and whether naturalized.

## 3 RESULTS

**Figure 1:**
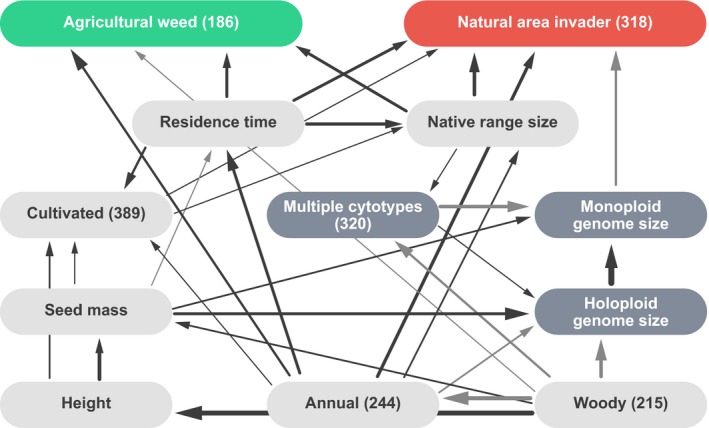
Bayesian network‐relating traits and introduction history to AW and NAI status for 688 angiosperms introduced to Australia generated using the *tabu* (modified hill‐climbing) algorithm implemented in the bnlearn package in R. Arrows indicate conditional dependence between variables, thickness magnitude, and shade (black = positive, gray = negative) direction of the relationship. Number of positives is given in parentheses below each categorical variable

Because the traits we used as predictors were correlated, we used phylogenetically confirmed Bayesian network (PBN, Appendix S1) to graph conditional probability dependencies between trait covariates on complete case data. Direct predictors of NAI in the PBN were in order of decreasing strength, native range size (+), annual life history (+), residence time (+), monoploid genome size (−), and whether cultivated (+), while those of AW were native range size (+), residence time (−), annual life history (+), and woody growth form (−) (Figure 1, Table S1). Residence time positively predicted cultivation, and both residence time and cultivation were positive predictors of native range size. This pattern of conditional dependencies may reflect shifts in geographic regions of origin as well as purpose and mode of entry of species over several centuries of angiosperm introductions. The strongest causal dependencies within the PBN were among and between morphological traits and genomic traits. Seed mass, maximum height, woody growth form, and annual life history are evolutionarily coordinated traits the values of which, as ensembles, result from allocation strategies and life history and physiological tradeoffs (Reich et al., 2003). As such, the strengths, but not necessarily the direction, of conditional probabilities connecting these variables are likely to be meaningful.

A key finding from PBN construction was that neither seed mass nor maximum height drives AW or NAI directly. While both traits served as good predictors of naturalized status in univariate regression analyses (below), this may be largely due to covariation with introduction history and/or genomic factors. Genomic traits were strongly linked within the PBN—species with large holoploid genome sizes tend toward large monoploid genome sizes and are less likely to form polyploids (Knight et al., 2005). Conditional probabilities also linked seed mass positively and woody growth form negatively to holoploid genome size, suggesting physiological or other constraints on genome size. The relative strength of monoploid genome size as an independent predictor of NAI, consistent with results of survival and regression analyses, is particularly interesting in that it suggests an important causal role.

**Figure 2:**
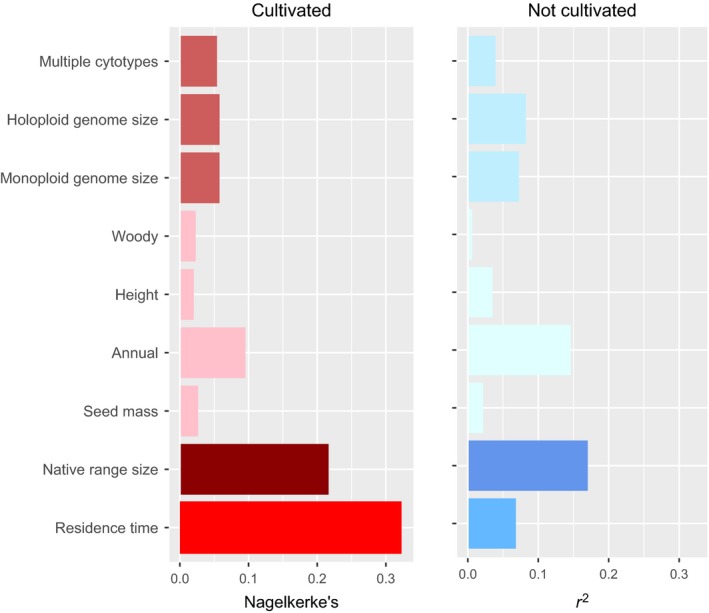
Bar plots of univariate GLM fits estimated by (Nagelkerke's *r* ^2^) by predictor and predictor type (genomic, functional/morphological, range size, and residence time), and whether cultivated (reds) or not (blues). Linear relationships to naturalization are negative for largest holoploid and for monoploid genome size and for seed mass

To gauge the magnitude of the contrasting effects of the full predictor data on naturalization status in cultivated versus uncultivated species and to capture potentially nonlinear univariate responses, we used GLM (logistic regression). Because we found the effects of phylogenetic nonindependence to be negligible in phylogenetic regression analyses (Table S2), we performed subsequent analyses without controlling for relatedness among taxa. When cultivated and uncultivated species were analyzed separately, residence time explained over four times the deviance in predicting naturalization in cultivated species than in uncultivated species. For species with residence times > 80 years, 89% of cultivated species, but only 68% of uncultivated species were naturalized. However, height, annual habit, monoploid, and largest holoploid genome size showed much larger effects in uncultivated than cultivated species (Figure 2). Thus, while residence time was by far the best predictor of cultivated species, in the absence of cultivation, native range size, genome size, and annual habit were better predictors of naturalization than residence time.

**Figure 3:**
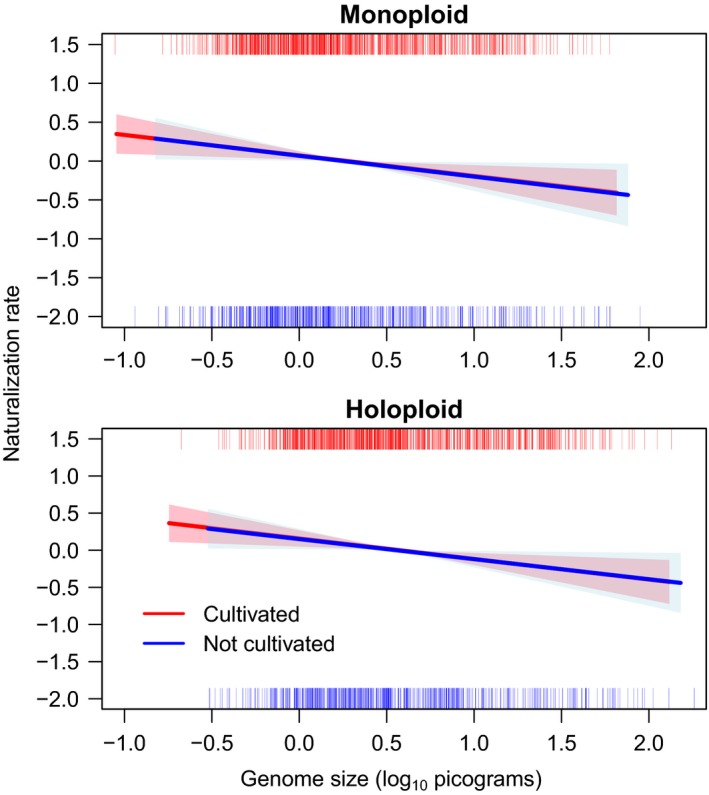
Rate of naturalization *y*‐axis as a function of monoploid and largest holoploid genome size, using spline fits of the Cox Proportional Hazard (survival package, R) contrasting cultivated (*n* = 904, 711) and uncultivated (*n* = 526, 404) angiosperms introduced to Australia. Residence time estimated from earliest Australian herbarium record (AVH 2015). Rugs at top and bottom of plots indicate the distribution of data, with intensity of color indicating the relative frequencies. Shaded regions represent 95% confidence intervals

**Figure 4:**
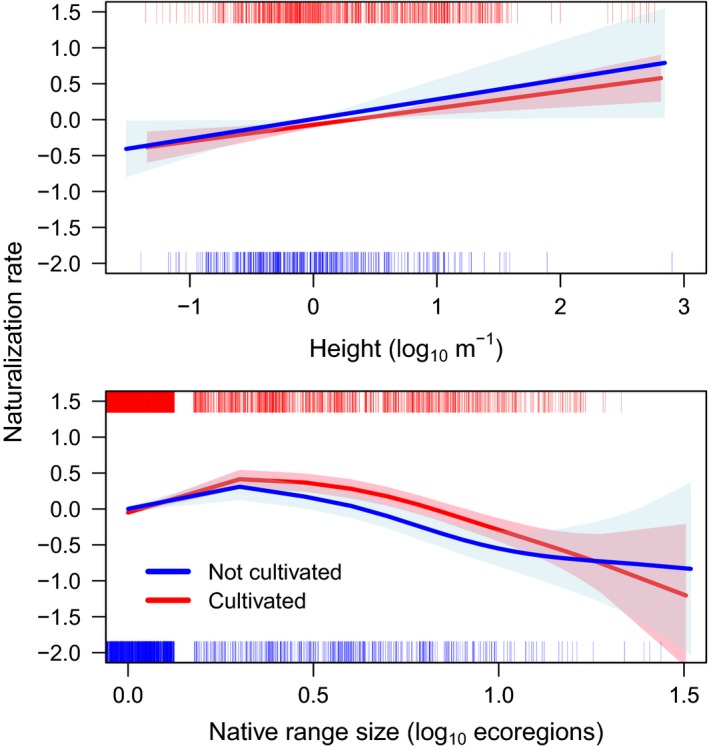
Rate of naturalization *y*‐axis as a function of height, and native range size, using spline fits of the Cox Proportional Hazard (survival package, R) contrasting cultivated (*n* = 710, 3,623) and uncultivated (*n* = 432, 1,502) angiosperms introduced to Australia. Residence time estimated from earliest Australian herbarium record (AVH 2015). Rugs at top and bottom of plots indicate the distribution of data, with intensity of color indicating the relative frequencies. Shaded regions represent 95% confidence intervals

**Figure 5:**
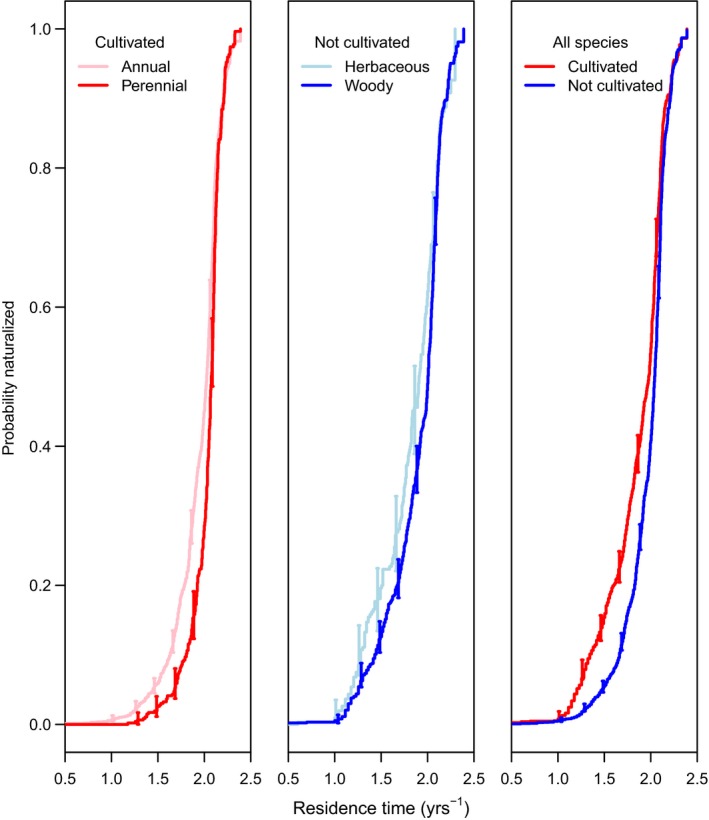
Kaplan–Meier plots showing increasing probability naturalized over time for contrasts in survival analyses (Cox Proportional Hazard) between annuals–biennials versus perennials (cultivated, *n *= 477, 2,205), herbaceous versus woody growth forms (not cultivated, *n* = 899, 315), and cultivated versus not cultivated (all species, *n* = 1,380, 339). Residence time estimated from earliest Australian herbarium record (AVH 2015). Vertical bars represent 95% confidence intervals. Note that annual–perennial contrasts were only significant for cultivated species and herbaceous–woody contrasts only significant in comparisons of uncultivated species

To further resolve the relationships between residence time, cultivation, and traits, we used survival analysis to incorporate time lags into models of the effect of traits on naturalization status while contrasting cultivated versus uncultivated species. For each trait, survival analysis was restricted to species for which residence time and trait predictor data were both available. All continuous predictors (aside from seed mass) showed significant relationships to rate of naturalization. Both species with and without a known history of cultivation, monoploid, and largest holoploid genome size exhibited a significantly (*p *<* *.04) negative linear relationship to the rate of naturalization (Figure 3). However, whether multiple cytotypes have been reported for a species was not a significant predictor of naturalization rate. Height was significantly positively associated with naturalization rate (Figure 4). For both cultivated and uncultivated species, naturalization rate increased as native range size increased from a single ecoregion (the native range size of 77% of species in the data set) to several ecoregions. Beyond this point, where a declining number of species are represented, naturalization rate decreased with increasing. Annuals exhibited a significantly higher rate of naturalization than perennials, but only in cultivated species. Herbaceous species showed a significantly higher rate of naturalization than woody species, but only in uncultivated species (Figure 5). The effect of cultivation was greater than any other binary predictor and doubled the rate of naturalization over some intervals (Figure 3). Yet, unlike in regressions, differences in trait effects on naturalization rates in cultivated versus uncultivated species were slight, possibly owing to much smaller sample sizes. In all survival analyses, confidence intervals were narrower for cultivated species, for which sample sizes were >1.5× larger.

Together, PBN**,** survival, and regression models explained a large amount of variation in naturalization status especially given data limitations. Moreover, as the data set was right‐censored—many species may come to be classified as naturalized eventually—estimated fits of the models are likely to be very conservative (Phillips, Murray, Leishman, & Ingram, 2010).

**Figure 6:**
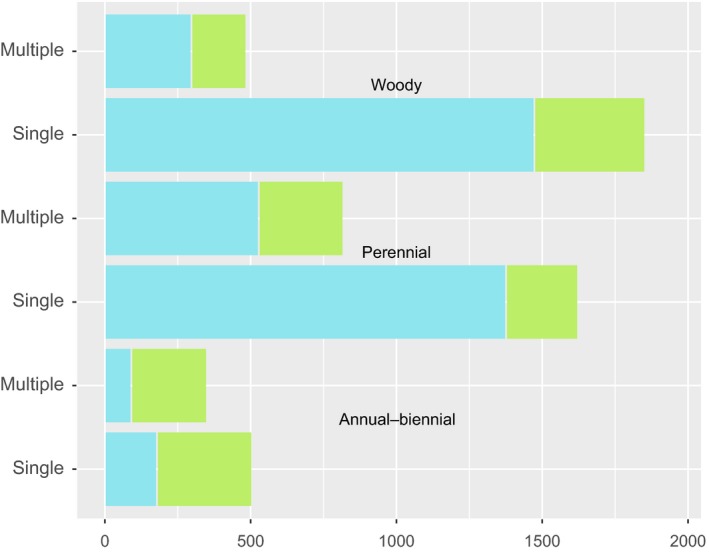
Fraction naturalized (green) is significantly higher (Fisher's Exact Test, *p* < .0001) among species for which multiple cytotypes have been reported whether woody (*n* = 2,338), herbaceous perennial (*n* = 2,441), or annual/biennial (*n* = 855)

Although not a significant predictor of naturalization in survival analyses, ploidal variation was a significant predictor of naturalized status in univariate regression models. Therefore, we tested whether the frequency of species with multiple cytotypes differed by naturalization status and growth form. The proportion of species for which multiple cytotypes have been reported was significantly higher (*p *<* *.0001; fisher.test in R; Fisher's Exact Test; Agresti, 1992) among naturalized species across all growth forms (Figure 6). Most introduced annuals–biennials (62%) have naturalized and the proportion of polyploid vs. diploid species that have naturalized is only slightly higher (74% vs. 65%). In contrast, only 15% of introduced herbaceous perennial and 13% of woody species have naturalized, and the proportion of polyploid species that have naturalized is roughly twice that of diploid species in either case (15% vs. 35% and 20% vs. 39%). Thus, the polyploid advantage appears more pronounced in species with longer generation times.

**Figure 7:**
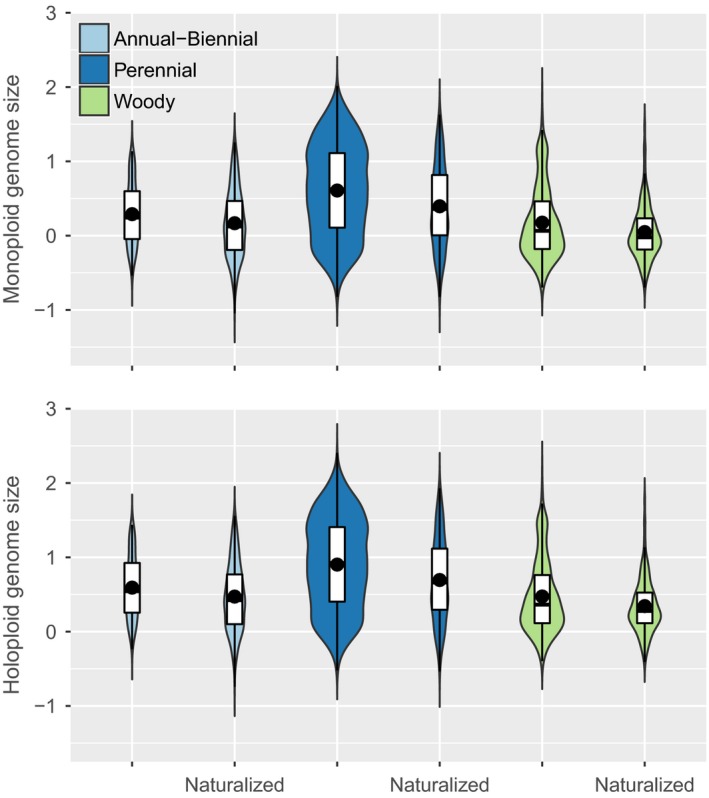
Violin plots showing distributions of monoploid (top) and largest holoploid (bottom) genome size (log_10_ picograms) by growth form (annual–biennial, perennial, and woody) and whether naturalized. The bottom and top of the boxes represent the first and third quartiles, points the means, dark bands medians, and colored shapes the probability densities of the genome size data. Differences between means for naturalized versus not naturalized within growth forms and differences between means by growth form were all significant at <0.03 after Bonferroni correction for multiple comparisons

We also tested whether genome size distributions differed by naturalization status and growth form and found genome size ranges broader, and means and medians higher in herbaceous perennials versus either annuals or woody perennials (Figure 7). While genome size means and medians were similar, although higher, in annual–biennials versus woody species, the genome size distribution of woody species was skewed toward lower values with a larger tail toward the high end. Across growth forms, naturalized species had lower mean genome sizes than species not naturalized. Within growth form classes, differences between monoploid and largest holoploid genome size means for naturalized versus not naturalized were significant (*p *<* *.03 after Bonferroni correction for multiple comparisons), as were differences in genome size means between growth forms. Importantly, the difference in mean genome size by naturalization status was larger in herbaceous perennials than in either annual–biennials or woody species.

## 4 DISCUSSION

Although residence time was the most important predictor of naturalization in regression analyses, and species with shorter generation times (annuals–biennials > herbaceous perennials > woody perennials) were more likely to have successfully naturalized, native range size was a stronger predictor of NAI than residence time. These results suggest that, given some minimum time threshold, physiological or adaptive features captured by native range size are more important than event‐level predictors. Moreover, residence time showed a strong positive conditional dependence on annual habit and weak negative dependence on seed mass in the PBN. Of the 5,125 species from Randall's database for which date of earliest herbarium record was available, new introductions following the initial colonization of Australia reached a peak roughly 70 years ago and have declined since, and the proportion of introductions that were annuals sharply decreased in recent decades. While the traits and origins of the species making up the introduced pool are likely to have changed over time, how representative this sample is of changing patterns in the total species pool cannot be determined. In the PBN, residence time also positively influenced naturalization via native range size and cultivation, suggesting additional introduction biases may have occurred over time. Curiously, residence time was much less important when predicting naturalization in uncultivated versus cultivated species in logistic regression, and, in survival analyses, the advantage of herbaceous over woody species was significant only for uncultivated species. This may be because data on past cultivation provides a good control for propagule pressure, making naturalization status as a function of generation length and residence time more predictable in cultivated species. In contrast, potentially large, but unknown, differences in propagule pressure in uncultivated species may obscure the effects of residence time and life history.

### 4.1 Genome size

Across analyses, the probability species had naturalized increased as genome size decreased. This was true after controlling for shared ancestry, after including the effect of residence time, and after incorporating covariance structure into models. In the PBN, monoploid genome size, strongly dependent on holoploid genome size, was the only biological trait directly predictive of NAI aside from native range size or covariates reflecting generation time (woody–herbaceous, annual–perennial). Moreover, the strength of the conditional dependence of NAI on monoploid genome size was marginally greater than that of annual habit or cultivation and 2/3 that of residence time (Table S1). However, agricultural weeds, typically annual species adapted to disturbance, did not show conditional dependence on genome size in the PBN. Interestingly, seed mass, height, and woody growth form, traits commonly used as predictors of naturalization or invasiveness in comparative studies (Pyšek et al., 2015; Schmidt, Springborn et al., 2012; Schmidt, Stephens et al., 2012), did not predict NAI directly. Rather, the primary effect of these traits on NAI may occur via constraints on genome size. Within growth forms, mean genome size was significantly lower in naturalized species. The difference in means was somewhat larger in perennials. However, perennials, naturalized or not, have a broader genome size distribution and larger mean genome size than annual or woody angiosperms such that mean genome size of naturalized herbaceous perennials is greater than the means for either annual or woody species that have not naturalized. Thus, while constraints on absolute genome size seem to be weaker in herbaceous perennials than in annual–biennials or woody perennials, the filter naturalization appears to exert on genome size is of a similar or greater relative magnitude.

In a review of the role of genome size in plant invasions, Suda et al. (2014) offer the broad generalization that monoploid genome size is linked to rate parameters (e.g., growth) and holoploid genome size to size parameters (e.g., seed mass). Although cell size is correlated with holoploid genome size, the rate of cell division is correlated with monoploid (per ploidy level) genome size (Bennett & Smith, 1976; Herben et al., 2012). Therefore, lower monoploid genome sizes may promote faster growth rates. In this way, the advantage of reduced genome size to naturalization may come primarily through direct controls on growth, fecundity, and survivorship, such that genome size should be viewed as a key functional trait as suggested by Knight et al. (2005) and Grime (1998). While Gallagher et al. (2011) found that holoploid genome size did not predict which acacias introduced to Australia had become invasive, survival analysis (Cox Proportional Hazard) of the same data, using year of earliest Australian herbarium record (AVH 2016) to estimate residence time, revealed a significant negative effect of log_10_ holoploid genome size (coef = −7.67, *p *=* *.02, *n *=* *91). Although the mechanisms are not resolved, these findings, collectively, suggest a central role for reduced genome size as a preadapted trait enabling introduced species to colonize and spread.

### 4.2 Polyploidy and genome size

Whereas Pandit et al. (2014) found genome size along with polyploidy to be important in predicting invasive members of a global pool of introduced species, we found the role of polyploidy to be weak and indirect. As such, our results concur with Herben et al. (2012) who found a negative relationship between genome size and regional abundance in herbaceous species native to central Europe with only a weak effect of ploidy. This is consistent with our finding that whether a species has known polyploid variants was not a direct predictor of naturalization in the PBN model. Rather, the effect of polyploidy may operate indirectly by leading to a reduction in monoploid genome size as suggested by Chen, Guo, and Yin (2010) and Leitch and Bennett (2004). Nor did species with polyploid cytotypes show significantly faster rates of naturalization in survival analyses, and the effect of polyploidy in regression models was weaker than that of genome size, particularly in uncultivated species. Nonetheless, successful angiosperm invaders included a significantly disproportionate number of taxa with polyploid forms. The absolute number of naturalized species with multiple cytotypes was greater than that of naturalized species with single cytotypes only in herbaceous perennials. This is likely because trees and shrubs tend not to form polyploids, perhaps due to physiological constraints on genome size (Ancel Meyers & Levin, 2006; Beaulieu, Leitch, Patel, Pendharkar, & Knight, 2008; Knight et al., 2005; Stebbins, 1938), and because the relative advantage of polyploids may be less important in rapidly reproducing annual–biennials. However, across growth forms, a disproportionate fraction of naturalized species included polyploid variants. Overall, genome size appears to impose a strong limit on the formation of polyploids, and the polyploid advantage in naturalization may be restricted to a relatively small range of genome sizes, growth forms, or environmental conditions.

### 4.3 Native range size

Native range size, not genome size, was the strongest predictor of naturalization in univariate regression analyses and was also a much more important predictor of NAI status than genome size in the PBN. Native range size (in this study measured by a tally of the number of different floristic regions in which a species is known to occur, and available for all species in the database) was meant to serve as a composite trait to capture climate generalism or physiological/morphological plasticity, but also, perhaps, a history of adaptation to human disturbance in the Eurasian context. Conditional dependence of native range size on residence time and cultivation in the PBN suggests that species with large native ranges enjoy an advantage that stems in part from earlier dates of introduction and a history of cultivation. When residence time was controlled for directly in survival analysis, the positive relationship with naturalization was restricted to the interval from one to three floristic regions, which took in 84% of species in the survival sample (*n *=* *1,142) and 98% of species in the full data set. (96% of species in the full data set and 76% of species in the native range size subset were native to a single floristic region.) However, the naturalization rate decreased at larger range sizes. Although, there was no strong evidence for this in preliminary analyses, statistical associations with native range size may stem from the identity of particular floristic regions with high phylogenetic diversity and, therefore, greater “genetic potential” as hypothesized by Fridley and Sax (2014). While the exact nature of the native range size effect is unclear, its importance in our results demonstrates that reduced genome size, as a factor promoting naturalization, functions in conjunction with a set of latent trait or geographic variables.

### 4.4 Data and model limitations

The aims of this study were broad, and the data elements of our analysis are necessarily coarse. We relied on expert opinion to define a broad set of overlapping naturalization classes that provide little information on the relative magnitude of ecological impact. We considered the entire continent of Australia leaving aside differences in climate matching between introduced species and the very distinct climate zones found there. We used date of the earliest herbarium record as a proxy for date of introduction, so that some residence times in our analyses are sure to be underestimated. A necessary simplification for our analysis was to consider genome size at the taxonomic level of species, using either monoploid genome size or holoploid genome size of the cytotype with the highest ploidy number, rather than by cytotype. We considered the occurrence of more than one cytotype as evidence for the potential of a species to undergo polyploidization. Species with data on genome size, ploidy, and seed mass were biased toward widespread and well‐studied species for which genome size and polyploid variants are more often documented—possibly inflating the importance of genomic factors. Alternatively, variation in the accuracy of the methods used to estimate genome size within the Kew database may have served to weaken the small genome size signal. Data on cultivation are likely to underestimate the number of species actually cultivated historically in Australia, which Randall (2007) estimates to be 97%, but may adequately capture species widely and commonly cultivated. Despite these limitations, our model revealed consistent patterns in the invasion success of plant introductions to Australia as a function of introduction history, genomic traits, native range size, and life history/growth forms.

### 4.5 Novel findings

Whereas the effect of functional traits on invasion success has generally been found to be weak relative to event‐level factors (see Peoples & Goforth), we found a relatively large role for traits even after controlling for phylogenetic relatedness. For example, although survival analysis revealed a naturalization advantage for cultivated species, in univariate logistic regression models, residence time was less important than other predictors (including genome size) in species not known to have been cultivated historically. Moreover, the effects of cultivation and residence time were similar to species‐level traits in PBN analysis, and, among traits, neither height and seed mass nor multiple cytotypes were direct predictors of naturalization. Critically, across all analyses of the largest publicly available data set on introduced plants we are aware of, and despite considerable noise, the effect of reduced genome size, while conditioned on growth form, was consistently important as a driver of successful naturalization.

### 4.6 Concluding remarks

Key elements in our analyses were the direct comparison of naturalization status across species documented as introductions to Australia, the inclusion of residence time estimates, the distinction between cultivated and uncultivated species, tests for the effect of phylogenetic relatedness, and the graphing of covariance structure. To expand and improve the detection of eco‐evolutionary drivers of invasion success, more flexible and comprehensive comparative approaches that merge phylogenetic controls with machine learning and statistical methods, such as those used in this study, are critical. These methods can elucidate patterns among evolutionarily linked traits (e.g., seed mass, height, specific leaf area, and genome size), and, in conjunction with much larger data sets that include introduction history, allow for more comprehensive models leading to more robust inferences. Understanding better how genome size as a single trait may integrate morphological features (including seed mass and growth form) and rates of adaptation to novel conditions appears critical to improvements in the screening and management of plant invaders.

## AUTHOR CONTRIBUTION

JPS compiled the data, performed the analyses, and drafted the manuscript. PRS assisted with phylogenetic analyses and edited the manuscript. JMD assisted with statistical analyses, suggested the figures, and edited the manuscript.

## CONFLICT OF INTEREST

None declared.
