# Supplementary material for: Residence time, native range size, and genome size predict naturalization among angiosperms introduced to Australia
Source: Ecol Evol. 2017 Oct 27;7(23):10289–300. doi: 10.1002/ece3.3505 (PMC5723587; doi:10.1002/ece3.3505)
Supplement: Supplementary file 1 [file ECE3-7-10289-s001.doc]

**Appendix S1**

**Phylogenetic Analyses**

**Methods**

DATA

Statistical inference in large comparative studies is generally complicated by phylogenetic non-independence of species as units of observation (Felsenstein 1985). Phylogenetic regression offers a means for testing and controlling for phylogenetic signal, but requires data and models from which evolutionary relationships can be estimated (Bloomberg *et al.* 2012). Since the genetic information necessary to generate a species-level tree was not available for most of the species in the data set, we used Phylomatic v3 (Webb & Donoghue 2005) to produce a phylogeny for analyses. The best resolved version of the Angiosperm Phylogeny Group base tree vIII (Stevens 2001; Bremer *et al.* 2009), a family level tree of Angiosperms, was used as the backbone tree. To estimate branch lengths, we used the BLADJ algorithm in Phylocom 4.2 (Webb *et al.* 2008) to interpolate distances based on existing dated trees (Wikström *et al.* 2001). Because sample sizes differed by predictor in univariate models, and complete case data (*n* = 688) was needed for the Bayesian network, we constructed separate trees for each analysis based on the set of species included. The resulting trees were only partially resolved to the species level, and like any supertree included information from a variety of trees of varying accuracy and estimated using varying data sources and methods. However, for the species compared we believe these trees represent the best phylogenetic estimates currently available. The fact that the results of phylogenetically confirmed regression analyses agreed with those using graphical models (see results below) also suggests that our overall qualitative conclusions are robust.

CONFIRMING GRAPHICAL MODELS GIVEN PHYLOGENETIC NON-INDEPENDENCE

As with regression, graphical models learned from multi-species data in comparative studies without incorporating the phylogenetic relationships among species may yield spurious results that fail to detect the actual causal structure among variables. For this reason, Shipley (2000) developed a method for confirming network structure given complex hierarchical patterns of non-independence within the data. These methods rely on testing *d-*separation (*d-*sep, Pearl 1988, Verma & Pearl 1988), the concept that variables unconnected by any edges (*d-*separated) are conditionally independent. Thus, for a graphical model to be valid, non-adjacent or *d-*separated variables, *X* and *Y*, must be independent, conditional on *Z*, the variable that separates them. However, apparent *d-*sep can be present in graphical models learned from data with dependencies (e.g. spatial, temporal, phylogenetic), because the algorithm used cannot control for non-independence within the data. However, networks learned in this way can be confirmed by 1) fitting, for each set of *d-*separated nodes, a mixed model to predict *Y* as a function of *Z* and all parent nodes of either non-adjacent variable, 2) calculating the probability (*p*_i_) that the partial regression coefficient associated with *X* is 0, 3) combining all the values of *p*_i_ via Fisher’s *C* statistic for the *k* *d-*separated variables in the model

*C* = *α* 2Σ(ln(*p*_i_)),

and 4) testing the significance of C (χ^2^ distribution with 2k degrees of freedom). The network model can be assumed to fit the data when C is not significant (p > 0.05), that is d-separated variables remain conditionally independent even when phylogenetic non-independence has been accounted for. The methods of von Hardenberg and Gonzalez-Voyer (2012) adapt Shipley's methods to deal with phylogenetic structure using phylogenetic generalized least squares (PGLS) regression. We, in turn, adjust the methods of von Hardenberg and Gonzalez-Voyer to include PGLM (phyloglm, phylolm package, R) in addition to PGLS (gls, nlme package, R) to test conditional independence of both continuous and binary dependent variables. If conditional independence is confirmed, the resulting model is a phylogenetically-confirmed Bayesian network (PBN).

PHYLOGENETIC LINEAR AND LOGISTIC REGRESSION

To assess the importance of covariates in predicting the naturalization status of species, we used *phyloglm* in the phylolm package (Ho & Ane 2014) in R (R Core Team 2016), which implements phylogenetic linear regression and phylogenetic logistic regression following the methods presented in Ives & Garland (2010). The *phyloglm* algorithm uses a parameter α to estimate the level of phylogenetic correlation in phylogenetic logistic regression analyses. α is analogous to the λ parameter in a phylogenetic generalized linear model (PGLM) analysis (see Revell 2010), and was used in all of our phylogenetic logistic regression analyses to adjust for the amount of phylogenetic signal observed in model residuals. Because sample sizes differed by predictor, we constructed

**Results**

PHYLOGENETIC CONFIRMATION OF THE BAYESIAN NETWORK

Using *d-*sep tests adapted from Pearl (1988) by Shipley (2000) and von Hardenberg & Gonzalez-Voyer (2013), we confirmed that, despite phylogenetic structure in the data, variables in the Bayesian network graph that were unconnected by any edges were conditionally independent (*n* = 688, *C* = 205.87, *k* = 14, *p* = 0.00001). Thus, results reflected a valid causal network among covariates that did not result merely from phylogenetic patterns in trait relationships.

PGLM MODELS

We used univariate PGLM models (Table S1) to assess the degree of phylogenetic signal in regression models predicting naturalization class by traits, and to estimate the relative importance of individual traits as predictors. We detected little phylogenetic signal (*α* < 0.04) across predictor and naturalization classes. In these models, the best predictors of NAI and AW were native range size (*r^2^* = 0.22, *r^2^* = 0.19, *n* = 9644), residence time (*r^2^* = 0.20, *r^2^* = 0.19, *n* = 4949), cultivation (*r^2^* = 0.14, *r^2^* = 0.09, *n* = 9644), and annual life history (*r^2^* = 0.10, *r^2^* = 0.16, *n* = 9816). After annual life history, monoploid genome size was the most important trait predict NAI (*r^2^* = 0.08, *n* = 2133); AW by height (*r ^2^*= 0.07, *n* = 1763). Woody growth form negatively, and annual life history positively, predicted both NAI and AW. While patterns were generally similar, cultivation explained less, annual life history more, variation in AW than NAI, and seed mass (*r^2^* = 0.01, *r^2^* = 0.03, *n* = 7200) and height were more important predictors of AW.

**References**

S. P. Blomberg, J.G. Lefevre, J.A. Wells, M. Waterhouse, Independent contrasts and PGLS regression estimators are equivalent, *Systematic Biology*, 2012, syr118.

B. Bremer, K. Bremer, M. Chase, M. Fay, J. Reveal, D. Soltis, P. Soltis, P. Stevens, An update of the Angiosperm Phylogeny Group classification for the orders and families of flowering plants: APG III, *Botanical Journal of the Linnean Society*, 2009, 161, 105, 121.

J. Felsenstein, Phylogenies and the comparative method, *American Naturalist*, 1985, 125, 1, 15.

L. S. T. Ho, C. Ane, A linear-time algorithm for Gaussian and non-Gaussian trait evolution models Systematic Biology, *2014*, 63, 397, 408.

A. R. Ives, T. Garland, Phylogenetic logistic regression for binary dependent variables, *Systematic Biology*, 2010, 59, 9, 26.

R Core Team, R: A language and environment for statistical computing, R Foundation for Statistical Computing, 2016, Vienna, Austria. <https://www.R-project.org/>.

L. J. Revell, Phylogenetic signal and linear regression on species data, *Methods in Ecology and Evolution*, 2010, 4, 319, 329.

B. Shipley, Confirmatory path analysis in a generalized multilevel context, *Ecology*, 2009, 90, 363, 368.

P. F. Stevens, Angiosperm phylogeny website, 2001, <http://www.mobot.org/MOBOT/research/APweb/welcome.html>

T. Verma, J. Pearl, Causal networks: semantics and expressiveness. Pp. 352–359 in Proceedings of the 4th workshop on uncertainty in artificial intelligence, 1988, Mountain View, CA. Schachter, R., Levitt, T. S., and Kanal, L. N., eds. Uncertainty in artificial intelligence, Vol. 4. Pp. 69–76. Elsevier, Amsterdam.

A. von Hardenberg, A. Gonzalez-Voyer, Disentangling evolutionary cause-effect relationships with phylogenetic confirmatory path analysis, *Evolution*, 2013, 67, 378, 387.

C. O. Webb, M.J. Donoghue, Phylomatic: tree assembly for applied phylogenetics, *Molecular Ecology Notes*, 2005, 5, 181, 183.

C. O. Webb, M.J. Donoghue, Phylomatic: a database for applied phylogenetics, 2002, <http://www.phylodiversity.net/phylomatic>.

N. Wikström, V. Savolainen, M.W. Chase, Evolution of the angiosperms: Calibrating the family tree, *Proceedings of the Royal Society of London, B, Biological Sciences*, 2001, 268, 2211, 2220.

**Table S1.** Conditional probabilities generated from Bayesian network (*tabu* algorithm implemented in the bnlearn package in R) relating traits and introduction history to AW and NAI status for 688 angiosperms introduced to Australia generated. Boldface variables are conditional on the variables (effects below them) in the following row. All continuous variables were log10-transformed.

| **Years since introduction** |  |  |  |  |
| --- | --- | --- | --- | --- |
| Seed mass | Annual |  |  |  |
| -0.0294 | 0.1254 |  |  |  |
|  |  |  |  |  |
| **Cultivated** |  |  |  |  |
| Years since introduction | Seed mass | Mature height | Annual |  |
| 0.2313 | 0.0563 | 0.1451 | 0.1302 |  |
|  |  |  |  |  |
| **Native range size** |  |  |  |  |
| Years since introduction | Cultivated | Annual |  |  |
| 0.2333 | 0.0684 | 0.1035 |  |  |
|  |  |  |  |  |
| **Seed mass** |  |  |  |  |
| Mature height | Woody |  |  |  |
| 0.7747 | 0.3743 |  |  |  |
|  |  |  |  |  |
| **Holoploid genome size** |  |  |  |  |
| Seed mass | Multiple cytotypes | Annual | Woody |  |
| 0.0881 | 0.0954 | -0.1596 | -0.4171 |  |
|  |  |  |  |  |
| **Monoploid genome size** |  |  |  |  |
| Seed mass | Holoploid genome size | Multiple cytotypes |  |  |
| 0.0206 | 0.8870 | -0.0983 |  |  |
|  |  |  |  |  |
| **Multiple cytotypes** |  |  |  |  |
| Native range size | Woody |  |  |  |
| 0.1821 | -0.1759 |  |  |  |
|  |  |  |  |  |
| **Mature height** |  |  |  |  |
| Woody |  |  |  |  |
| 0.8471 |  |  |  |  |
|  |  |  |  |  |
| **Annual** |  |  |  |  |
| Woody |  |  |  |  |
| -0.4514 |  |  |  |  |
|  |  |  |  |  |
| **Natural area invader** |  |  |  |  |
| Years since introduction | Monoploid genome size | Native range size | Cultivated | Annual |
| 0.2421 | -0.1604 | 0.3904 | 0.1575 | 0.1347 |
|  |  |  |  |  |
| **Agricultural weed** |  |  |  |  |
| Years since introduction | Native range size | Cultivated | Annual | Woody |
| 0.2332 | 0.2402 | 0.0897 | 0.1635 | -0.1143 |

**Table S2.** Results of univariate phylogenetic logistic regression models (phylolm package, R) predicting NAI and AW among angiosperms introduced to Australia. For each class, predictor variables are ranked from greatest to least pseudo-***r^2^*** value. Sample size (*n*) indicates total number of records (right) and number of positives for each class (left). *α* is a measure of phylogenetic signal.

| **Natural area invader** | | | | | | | |
| --- | --- | --- | --- | --- | --- | --- | --- |
|  | | ***n*** | | **Coef.** | ***p*** | ***α*** |  |
| **Multiple cytotypes** | | 9016 | 451 | 1.06 | <0.00001 | 0.02 | |
| **Holoploid genome size** | | 2911 | 581 | -0.92 | <0.00001 | 0.02 | |
| **Monoploid genome size** | | 2133 | 653 | -0.91 | <0.00001 | 0.03 | |
| **Woody** | | 9745 | 1,619 | 1.16 | <0.00001 | 0.01 | |
| **Height** | | 1763 | 475 | -0.26 | <0.00001 | 0.02 | |
| **Annual** | | 9816 | 1,440 | 1.65 | <0.00001 | 0.02 | |
| **Seed mass** | | 7200 | 1,272 | -0.15 | <0.00001 | 0.02 | |
| **Native range size** | | 9644 | 1,893 | 4.89 | <0.00001 | 0.02 | |
| **Cultivated** | | 9644 | 1,893 | 1.54 | <0.00001 | 0.01 | |
| **Residence time** | | 4949 | 1,695 | 2.27 | <0.00001 | 0.02 | |
|  |  | | | | | | |
| **Agricultural weed** | | | | | | | |
|  | | ***n*** | | **Coef.** | ***p*** | ***α*** |  |
| **Multiple cytotypes** | | 9016 | 722 | 1.28 | <0.00001 | 0.02 | |
| **Holoploid genome size** | | 2911 | 312 | -0.43 | <0.00001 | 0.02 | |
| **Monoploid genome size** | | 2133 | 243 | -0.54 | <0.00001 | 0.02 | |
| **Woody** | | 9745 | 821 | 0.68 | <0.00001 | 0.02 | |
| **Height** | | 1763 | 267 | -0.68 | <0.00001 | 0.02 | |
| **Annual** | | 9816 | 732 | 2.15 | <0.00001 | 0.02 | |
| **Seed mass** | | 7200 | 698 | -0.14 | <0.00001 | 0.02 | |
| **Native range size** | | 9644 | 932 | 4.18 | <0.00001 | 0.02 | |
| **Cultivated** | | 9644 | 932 | 1.54 | <0.00001 | 0.02 | |
| **Residence time** | | 4,949 | 874 | 2.93 | <0.00001 | 0.02 | |
